# Clients’ Experiences of a Community Based Lifestyle Modification Program: A Qualitative Study

**DOI:** 10.3390/ijerph6102608

**Published:** 2009-10-02

**Authors:** Ruth S.M. Chan, Kris Y.W. Lok, Mandy M.M. Sea, Jean Woo

**Affiliations:** 1 Department of Medicine & Therapeutics, Chinese University of Hong Kong, Hong Kong, China; E-Mails: kris.lok@cuhk.edu.hk (K.Y.W.L.); jeanwoowong@cuhk.edu.hk (J.W.); 2 Centre for Nutritional Studies, Chinese University of Hong Kong, Hong Kong, China; E-Mail: mandysea@cuhk.edu.hk

**Keywords:** lifestyle modification, weight management, obesity, patient-centered care, qualitative

## Abstract

There is little information about how clients attending lifestyle modification programs view the outcomes. This qualitative study examined the clients’ experience of a community based lifestyle modification program in Hong Kong. Semi-structured interviews were conducted with 25 clients attending the program. Clients perceived the program had positive impacts on their health and nutrition knowledge. They experienced frustration, negative emotion, lack of motivation, and pressure from others during the program. Working environment and lack of healthy food choices in restaurants were the major perceived environmental barriers for lifestyle modification. Clients valued nutritionists’ capability of providing professional information and psychological support in the program. Our results suggest that nutritionist’s capability of providing quality consultations and patient-centered care are important for empowering clients achieve lifestyle modification.

## 1. Introduction

Obesity is posing a growing threat to public health throughout the world and places extra burden on health costs. According to the Population Health Survey conducted in 2004 in Hong Kong, 38.8% of population was overweight and 21% were obese [1]. These figures of overweight in adulthood have doubled since 2003. This is a major cause of concern as obesity is often related to other health risks such as cancer, heart disease, diabetes, dyslipidemia, hypertension and stroke. While prevention is clearly important, effective management of those with obesity continues to be a public health challenge.

Common management options consist of lifestyle modification in terms of diet and physical activity, pharmacological methods, and surgery for the morbidly obese [2]. It has been proposed that a weight management and treatment algorithm should consist of diet, exercise and behavioral therapy. As a result, numerous commercial and proprietary weight loss programs have been introduced [3]. Studies have shown that crucial to the success of most programs is the inclusion of a component of behavioral modification [2,4].

Most commercial weight loss programs in Hong Kong offer a range of options from very low calorie exchange diets, meal replacement products, supplements or unbalanced meals. Often these may be unaffordable for many people or carry claims of unrealistic weight loss. The Lifestyle Modification Program (LMP) examined in this study is a self-help community based program in Hong Kong for those who are overweight or obese. It provides low calorie exchange diets, physical and behavioral weight control methods individualized by nutritionists during counseling sessions. The purpose of the LMP is to develop lifestyle behaviors that encourage increasing caloric expenditure while decreasing caloric intake, with an emphasis on long term lifestyle and behavior change. Cognitive behavior therapy (CBT), which is derived from cognitive and behavioral psychological models of human behavior that include theories of normal and abnormal development, and theories of emotion and psychopathology, is applied as the strategy on behavior modification in the LMP [5]. Five levels of influence were identified by McLeroy and colleagues [6] for health-related behaviors and conditions. These levels include: (1) *intrapersonal* or *individual* factors; (2) *interpersonal* factors; (3) *institutional* or *organizational* factors; (4) *community* factors; and (5) *public policy* factors. The LMP highlights the importance of collaboration between clients and nutritionists to identify and understand the problems in terms of relationship between thoughts, feelings and behavior, to find out solutions, and to test and re-test those solutions is important for cognitive and behavior changes. The LMP therefore focuses on exploring the intrapersonal factors and the interpersonal factors that influence the clients to make lifestyle changes. Intrapersonal factors, such as knowledge, attitudes, beliefs, and personality traits; and interpersonal factors, such as the social network and social support systems are identified by the LMP nutritionists for cognitive and behavior changes [6]. A process of change may be involved, in which clients adjust their attitudes to the problems, consider the consequence of the problems and assess self-confidence in relation to a possible change [7]. The LMP also highlights the use of patient-centered approach in the counseling process. Patient-centered approach can be defined as involving the health professional trying to understand how the patients view their problems, and working on potential solutions together to culminate in the patient feeling they have developed their own realistic plans for change [8]. Five main domains are advocated in the patient-centered approach, which include (1) exploring the patients’ main reason for the visit, concerns, and need for information; (2) seeking a comprehensive understanding of the patient’s personal, developmental and family issues; (3) finding common ground on what the problem is and mutually agrees on management plan; (d) enhancing risk reduction and health promotion; and (e) enhancing the continuing relationship between the patient and the therapist [9,10].

As lifestyle modification is a complex process, which involves both behavioral and cognitive changes [11], multiple theories are combined in the CBT of the LMP. These theories include the Health Belief Model [12], which addresses the individual’s perceptions of the risk posed by a health problem (susceptibility, severity), the benefits of avoiding the risk, and factors influencing the decision to act (barriers, cues to action, and self-efficacy), the Stages of Change Model [13], which describes individuals’ motivation and readiness to change a behavior, the Social Cognitive Theory [14], which describes a dynamic, ongoing process in which personal factors, environmental factors, and human behavior exert influence upon each other, and the Consumer Information Processing Theory [15], which postulates that information must not only be available but also believed to be useful and format-friendly to the consumer.

The effectiveness of lifestyle modification for the treatment of obesity has been assessed recently [4,16]. However, most studies focus on measurable health outcomes, such as weight loss and change in body fat percentage, and there is little information about how clients attending lifestyle modification programs view the outcomes and how such programs impact upon their lifestyle and behavior. Although it has been recognized that the views of the client should be included in outcome measurement [17], little information is available in the literature related to client involvement in evaluating the outcomes of lifestyle modification programs [18,19]. The purpose of this study was to obtain views of clients attending a community based LMP on lifestyle and behavior change, in order to facilitate continuing development of the whole service in supporting individuals to make sustainable lifestyle changes.

## 2. Methods

### 2.1. The LMP Components

The LMP is carried out at the Centre for Nutritional Studies, The Chinese University of Hong Kong. The LMP was established in 2002 and over 6,500 clients have attended the program. The LMP design is adopted from the experience of previous study [2]. As mentioned earlier, lifestyle modification involves both behavioral and cognitive changes [11]. The LMP is developed based on the CBT concept and multiple theories.

Upon enrollment in the program, all individuals undergo a comprehensive health and dietary assessment; each individual completes an initial nutrition assessment with a nutritionist. During the initial assessment, the nutritionists carry out a complete behavioral assessment and collect important information, such as client’s history of health problems, current eating and lifestyle patterns, specific eating-related behaviors, knowledge of risks associated with current eating patterns, concerns and feelings about specific lifestyle changes (perceived susceptibility and perceived severity), past experiences and barriers to change in dietary intake and lifestyle (perceived barriers), reasons for changing current eating patterns (cues to action), and readiness to change (stage of change). The nutritionists also discuss the expected duration and specific dietary and lifestyle advices to achieve a desirable weight status with the clients. Thereafter, an exercise instructor designs individualized cardiovascular and resistance exercises to perform at home. On completion of the nutrition assessment and exercise advice, clients are encouraged to attend weekly, 15 to 20 minute counseling sessions with the same nutritionist and exercise instructor, respectively.

During the follow up counseling, the nutritionists review client’s compliance on diet, exercise adherence and progression. The nutritionists review food records to ensure nutritional adequacy of the diet and offer recommendations for controlling caloric intake. The dietary component and portion sizes of the program are based on American Dietetic Association, with 20% caloric restriction as appropriate to the individual [20]. A varied balanced diet with an emphasis on fruit and vegetables, and low-fat, low- glycaemic index and low calorific products in appropriate portions are encouraged. Clients are encouraged to increase daily activity, accumulating 30 to 60 minutes of cardiovascular activity 1 to 5 days/week. Clients can repeat these exercise consultations as often as they feel necessary. Other than reviewing client’s compliance on diet and exercise adherence, the nutritionists encourage the clients to share any personal and environmental barriers to lifestyle change. The nutritionists also assess the client’s feelings about the dietary advice and the progress, provide ongoing support and encouragement for the clients, re-define goals based on client’s feeling and progress, and affirm client’s efforts and achievements to enhance client’s self-efficacy. As to minimize the barriers to lifestyle changes, each client is provided with a booklet for portion size exchange and tips for eating out. The nutritionists also encourage the clients to bring new food products and restaurant menu to discuss and explore healthy choices, and to send their enquiries to the nutritionists by email or phone. Moreover, regular case meetings are organized among the nutritionists to exchange case management skills and updated information on nutrition, lifestyle and health.

### 2.2. Study Participants

The participants were clients attending the LMP. Clients who were overweight [with body mass index (BMI) between 23 and 24.9 kg/m^2^] or obese (with BMI of at least 25 kg/m^2^) based on the World Health Organization’s criteria for Asians [21], and who were not on any prescription medication were included in this study. They were informed of the study when they attended their appointment, and recruited on a voluntary basis. No reward was given for their participation. Those who agreed to participate were interviewed by one health researcher (RC) and one dietitian (KL) not involved in the treatment of the participants. Informed consent was obtained from all participants. The study was approved by the Ethics Committee of the Chinese University of Hong Kong.

### 2.3. Procedures and Data Analysis

A semi-structured interview guide was used to collect participants’ views in individual interviews. Topics included their previous number of attempts to lose weight and the outcomes, their progress and overall experience in the LMP, perceived barriers for following the LMP advice, ways that the nutritionists helped them overcome such barriers, their views on the nutritionists, and the perceived impacts of the LMP on their lifestyle and behaviors. Personal details including age, gender, socioeconomic status, duration since joining the LMP, and weight status at the start of LMP and at the time of interview were collected. The interviews lasted between 15 and 30 minutes. Each interview was recorded and transcribed verbatim. Transcripts were reviewed twice to check accuracy and detail of transcription. The transcripts were read by each interviewer. Key words and phrases in the transcripts were identified, compared and formed into main themes and subthemes using method described by Ryan and Bernard [22], which were discussed and organized together by the interviewers (RC & KL). A total of 25 subjects were approached to join the study and all consented to participate. A final sample of 25 subjects were used as no new information emerged from the last few interviews of each round indicating that saturation had occurred [23].

## 3. Results

### 3.1. Participant Characteristics

Characteristics of participants are presented in Table 1. There were 21 female and four male participants. The participants aged between 18 and 62 years. All except one were ethnic Chinese. Their duration since joining the LMP ranged from one month to two years and nearly two-third of them had tried to lose weight before (n = 17), using regimes consisting of dietary restriction or diet replacements (such as milk shake supplement), doing exercise and using the over-the-counter weight loss pills.

### 3.2. Perceived Impact of the LMP on Health and Satiety

Based on the data emerging from the interviews, all the themes and subthemes identified are shown in Table 2.

Most (n = 18) valued the LMP as a good program for lifestyle modification and claimed that the LMP provided a more healthy way of lifestyle modification and weight management. Some (n = 8) claimed that LMP did not have any side effect on their health and some (n = 6) claimed that there was a lower chance of weight rebound in the LMP as compared with other weight loss methods.

The LMP seems to be healthier compared to the treatment by the weight reducing machines. Some of my friends showed weight rebound after stopping the treatment by the weight reducing machines

(No. 14:34).

Very healthy...throughout the LMP, you do not need to worry about any side effects and it is very healthy

(No. 16:27).

Apart from weight loss, participants also described that their health status improved after joining the LMP.

Surely I have lost weight and become thinner. My health is better now, at least my eye is less swollen and my joints are less painful than before

(No. 23:26).

After joining the LMP for three months, my weight has decreased from 70 kilograms to 60 kilograms and the blood cholesterol level has returned to normal

(No. 20:32).

Some participants (n = 5) were also surprised by the feeling of fullness from the diet plans in the LMP while they were losing weight.

As for weight loss, I suppose the diet plan does not allow you to eat fully. But it is not the case for the LMP. I do not understand its rationale. Following the LMP diet plan makes me feel full and I do not feel hungry

(No. 14:40).

I was surprised by the feeling of fullness experienced at the beginning of the LMP. I did not think that I ate less, but meanwhile I was able to lose weight gradually

(No. 22:24).

### 3.3. Perceived Impact of the LMP on Nutrition Knowledge and Self-Control of Food Intake

Participants (n = 5) commented that their nutrition knowledge was increased through the LMP and this enabled them to identify more healthy food choices.

In the past, I could not identify the healthy choices. I had no doubt that vegetables were healthy, so I ate vegetables regardless of the cooking methods. I also thought that vegetarian dishes were healthy, so I ate all kinds of vegetarian dishes. But now, I realize that there are many misconceptions. I know how to avoid such misconceptions and make healthy choices

(No. 24:50).

For example, I will try to choose less oily food. In the restaurant, I will try to avoid foods with sauce or I will choose foods with less fat

(No. 9:26).

Participants (n = 2) also described that the LMP could raise their nutrition and health awareness and increase their self-control of food intake.

I know how to choose the right foods now. In the past, although I knew that some foods might make me gain weight, I could not resist them. But now, I can resist them

(No. 17:28).

Apart from weigh loss, I now pay more attention to my general health. I know that losing weight could help me lower cholesterol level and body fat, which are important for long term health

(No. 24:68).

### 3.4. Perceived Barriers to Change – Psychological Factors

Several participants (n = 10) commented on psychological factors being one of the barriers to lifestyle and behavior change in the LMP. These included frustration, negative emotion, unstable mood, lack of motivation, lack of trust in their ability to succeed, and loss of confidence in nutritionist’s ability to provide effective intervention in the LMP. Such factors may be linked with occasional weight relapse and compulsive eating in the LMP.

In the LMP, you occasionally experienced a cycle of weight loss and rebound, which made you very upset. I asked myself why there was a weight rebound and felt very frustrated. Also, when you joined the LMP for a certain period, you became less self-disciplined

(No. 14:54).

I felt bored sometimes. Just imagine if you ate similar foods everyday, it was really a boring and hard process

(No. 18:68).

Sometimes, I was frustrated because my weight remained the same for such a long treatment period. At that moment, you started to doubt your own ability to succeed and your nutritionist’s ability to provide an effective intervention

(No. 18:64).

Some (n = 8) experienced pressure and lack of support from family, friends and colleagues. Pressure was linked with fear of not achieving dietary goals, lack of healthy food choices in restaurants and fear of discouraging remarks from others.

The most difficult thing comes from eating out with friends or colleagues. Each time when I see the nutritionist, I feel a bit stressful. I am afraid of weight rebound and not achieving the dietary goals. So I feel a bit stressful whenever I eat out with my friends or colleagues

(No. 11:42).

If all your friends choose a restaurant which you know you cannot find any healthy choices, it is difficult for you to reject their decision. You may reject occasionally but not always

(No. 18:116).

When you eat out with friends or colleagues and you restrict yourself to eating certain food choices, they will say “Oh no...not again!”

(No. 18:68).

I have low self-discipline. I need my family members and friends to remind me to self monitor. They can eat whatever they want, but they must keep those foods out of my sights

(No. 4:50).

### 3.5. Perceived Barriers to Change – Environmental Factors

Participants (n = 5) shared that the working environment was one of the environmental barriers for lifestyle and behavior change. Some found it difficult to carry out the LMP diet plans in the working environment while some had difficulties in carrying out exercise programs due to long working hours.

I tried to prepare noodles at home and bring it to my office. A refrigerator was however not available in my office, the noodles were easily spoiled at room temperature

(No. 22:32).

For me, it is not difficult to follow the diet plan. However, it is difficult to find time to do more exercise due to busy work

(No. 21:36).

Some participants (n = 7) also commented that lack of healthy food choices in the restaurants and the occasional special events (i.e. banquet) were the other environmental barriers for lifestyle modification.

The restaurants near my workplace offer limited healthy choices. Most dishes are served with unhealthy sauces and most are fried foods. I definitely cannot find any good choices. So I cannot follow the diet plan perfectly

(No. 19:32).

It’s difficult to find healthy choices in Hong Kong restaurants. If you want to have healthy choices, you often need to have meals at home or bring your home-made healthy dishes to the workplace. It is quite difficult to arrange sometimes

(No. 15:66).

Once if you go to a banquet or go travelling, you cannot follow the diet plan

(No. 9:24).

### 3.6. Client’s Perceptions of Nutritionist’s Roles in the LMP

All interviewees described that the nutritionists should not only give technical advice on lifestyle modification, but also should provide psychological support for them in the LMP. The dual role was what the interviewees expected a good nutritionist should be in the LMP.

If the nutritionist can provide some sorts of emotional counseling, ask me more about my feelings or difficulties, this would be better

(No. 25:108).

If the nutritionist has more understanding of your thoughts and difficulties and she/he is able to help you solve such difficulties, it will be better than just giving you a menu and asking you to follow

(No. 14:62).

#### 3.6.1. Nutritionist being an information source in the LMP

Near half of the participants (n = 12) valued the LMP nutritionists as a good information source. They commented that the nutritionists were knowledgeable and professional in giving clear and practical dietary advice. They also described that the nutritionists were flexible and open minded.

For some special events, my nutritionist will adjust my diet plan and give me some special tips. For example, in case of hot pot meal (Chinese style of Fondue), she teaches me to eat vegetables first to give me feeling of fullness. So I know how to choose foods in special events

(No. 6:88).

The diet advice taught by my nutritionist is practical and clear. For example, the size of the forefinger plus the middle finger is similar to one portion of meat, and one medium bowl of rice contains three portions of carbohydrates. So these examples are easy to be applied in my daily life

(No. 8:138).

If I cannot follow the diet plan, my nutritionist will encourage me and set some easier goals for me to achieve

(No. 5:62).

During the LMP, my nutritionist offered me flexibility in eating snacks. She wouldn’t advise me to stop eating all snacks. Instead, she allowed me to reduce snack consumption gradually. This made me feel better

(No. 19:44).

My nutritionist is willing to discuss new products with me. She will ask me to bring them back in the next appointment and explore what they are. She is willing to accept new things

(No. 14: 126).

#### 3.6.2. Nutritionist being a psychological supporter in the LMP

Majority (n = 19) of the participants valued the psychological support from the nutritionists in the LMP. The psychological support from the nutritionists included on-going encouragement, patience, non judgmental attitude, acceptance and showing understanding.

Sometimes I cannot follow the diet plan, she (the nutritionist) would not give me any negative comments. Instead, she would explain to me that the weight loss would be smaller than expected

(No. 17:48).

If I was unable to follow the diet plan, she (the nutritionist) would adjust the diet plan based on my job nature, my personality and my recent status. She would not say something negative or discouraging to me. She instead showed her understanding and this made me feel better and encouraging

(No. 5:80).

When my weight cannot meet the targeted weight, she (the nutritionist) would kindly ask me if I had any difficulties in following the diet plan

(No. 8:124).

She (the nutritionist) would comfort me when I felt frustrated because of not achieving the weight loss target. She (the nutritionist) would explain the reason to me and encourage me

(No. 14:68).

Apart from talking about diet and health issues, some (n = 3) participants commented that the nutritionists were willing to listen to their sharing on personal feelings and family matters. They could share with the nutritionist as friend.

She (the nutritionist) would talk with me on my school life and daily life

(No. 4:78).

We share as friends. I sometimes share my unhappiness with her (the nutritionist). She will comfort me. It’s more important than just giving you diet menu

(No. 14:74).

### 3.7. Improvements for the LMP

Although most participants valued the efforts made by the nutritionists, some (n = 11) commented that the consultation time could be longer and there were some nutritionists’ variations in counseling skills in the LMP. They expected that the nutritionist could spend more time on understanding their habitual diet pattern and allowing them to ask more questions on the diet plan.

I have around ten minutes for each consultation. When I have some questions on diet, I need to ask these questions hurriedly within the ten minutes’ time. If the consultation time is longer, it would be better

(No. 19:46).

I think different nutritionists have different working and presentation styles. Nutritionist A is more active in explaining the diet plan to me whereas nutritionist B is relatively passive. She will only explain more to you if you ask her

(No. 15:78).

## 4. Discussion

Our results suggested that clients perceived the LMP had positive impacts on client’s health, nutrition and health knowledge. Meanwhile, the findings showed that clients experienced both psychological and environmental barriers for lifestyle and behavior change throughout the LMP, and the nutritionists’ capability of providing professional information and psychological support was important element for helping clients overcome such barriers.

Lifestyle modification is a complex process, which involves both behavioral and cognitive changes [11]. A process of change may be involved, in which clients adjust their attitudes to the problems, consider the consequence of the problems and assess self-confidence in relation to a possible change [7]. The cognitive behavior therapy emphases the collaboration between clients and nutritionists to identify and understand the problems in terms of relationship between thoughts, feelings and behavior, to find out solutions, and to test and re-test those solutions is important for cognitive and behavior changes [5]. This study identified several psychological factors and environmental factors as barriers to lifestyle modification. Psychological barriers, including frustration, negative emotions, lack of trust in own ability to succeed, loss of confidence in the nutritionist’s ability to provide an effective intervention, pressure from others; and environmental barriers from workplace and restaurants described by our clients, were in line with previous studies [24–26]. Jones *et al.* collected obese adults’ views on the dietetic service in West of Scotland for weight management in a qualitative study. Similar psychological factors were identified as to our findings. These factors included depression, frustration, negative emotions, stress, lack of motivation, hating to feel committed, and a lack of trust in their ability to succeed or monitor themselves. Although our study and Jones’ study identified a lack of healthy eating facilities and meals out in restaurants as environmental barriers to lifestyle changes, Jones’ study also suggested other factors as barriers to lifestyle change. These factors included cost of healthy food choices and physical elements, such as age, medical problems, and food allergies and intolerances, which were not reported by the clients in our study [25]. However, as with our study, pressure from others and lack of social support were identified as one of the important barriers to lifestyle changes in obese people in different cultural settings [25,28].

Most participants commented that the LMP had positive impacts on their health and nutrition knowledge. These impacts are essential for lifestyle modification as people would change behavior if they come to believe that the change is both of value and achievable [27]. The resulting improvements in health and physical function reported in this study and previous studies [28,29] were regarded as important motivators for lifestyle modification. These improvements included dietary-related problems, such as reduced cholesterol and reduced blood pressure, decreased clothes size, and feeling lighter as reported in our study and previous studies [25,29]. Previous studies also identified improvements in mood, self-esteem, confidence and social lives as other motivators for lifestyle change [25,29].

The LMP clients commented that a good nutritionist should be able to give clear and practical advice and psychological support. These comments were supported by previous work that health professionals should avoid prescribing healthy lifestyles that a patient should follow, but should act as a coach to encourage patients to explore their motivations, and to empower them develop their own healthy lifestyles [30,31]. Findings of previous studies comparing the effectiveness between a lifestyle modification program and a standard dietetic care also suggested that the inclusion of behavioral change approach and motivational interviewing on top of just giving diet and exercise advice was important for weight management and maintenance [2,32]. Patient-centered approach and various behavioral change techniques, such as expressing empathy, supporting self-efficacy and exploring client’s readiness to change, were the key elements of a lifestyle modification program [32,33].

The outcomes of behavioral treatment are influenced by the behavior of the health professional [34], and there is evidence from an obesity treatment program showing that only those dietitians with the appropriate interpersonal skills, expertise and training should undertake weight management program [33]. Training in techniques, including reflective listening skills by using reflective statements [30,35], skills for new adaptive learning and management relapses [8], techniques for involving the patient in active problem solving [30] and building up a collaborative relationship with client [30], are important for facilitating behavioral change [33]. Our results indicated that the LMP nutritionists were able to integrate different behavior change theories in the counseling process, and were generally able to fulfill the clients’ expectation of being a good nutritionist. Most participants in this study valued the LMP nutritionists as a good information guide, and being able to give clear and practical dietary advice. These comments were in line with the Consumer Information Processing Theory, which postulates that information must not only be available but also believed to be useful and format-friendly to the consumer [15]. Our clients also commented that the LMP nutritionists were flexible in adjusting diet plans and refining goals tailored to the client’s situation, and they valued the psychological support, such as on-going encouragement, patience, and acceptance from the LMP nutritionists. These techniques may increase client’s self-efficacy in making behavioral changes, which reflected the integration of the Social Cognitive Theory in the counseling process. The Social Cognitive Theory states that if individuals have a sense of self-efficacy, they can change behaviors even when faced with obstacles. If they do not feel that they can exercise control over their health behavior, they are not motivated to act, or to persist through challenges [14]. Findings of a previous study also showed that clients valued the counseling skills of dietitian. In a qualitative study exploring obese adults’ views on the dietetic service, interviewees valued the professional support, motivation and encouragement that they received from the dietitian, and described that the dietitian was “somebody there that would listen to you”. The interviewees also commented that the dietary advice was practical and easy to follow [25].

The importance of patient-centered care has been increasingly advocated for health care practice [36,37]. There is also evidence that involving patients in decision making can improve a variety of health outcomes in diverse settings [38,39]. Our data showed that clients came across a wide spectrum of psychological and environmental barriers in the LMP, but their appreciation on the technical and psychological support from the nutritionists highlighted the value of patient-centered care offered in the LMP. However, similar to previous findings, time constraint and staff skillfulness in delivering consultations were claimed by our clients as main factors influencing client understanding and consequent satisfaction [37,40]. Previous studies examining the relationship between consultation length and outcomes in general practice showed that longer consultations were significantly associated with better recognition and handling of problems, and with better patient enablement [41,42]. However, it has been suggested that patients’ perceptions of consultation length were influenced not just by actual consultation length, but by their experience (i.e. the quality) of the consultations [43]. Therefore, it has been suggested that the focus of quality should shift from ‘how much time’ to ‘how best to use that time’ [44].

There are limitations in this study. Subjects in this study were recruited on voluntary basis and they may be prone to have more positive views about the LMP. However, the interviewers recruited these subjects on random days and consultation slots, which were unknown to the nutritionists. This could help minimize the sampling bias in this study. However, the views collected in this study may not be generalized to the general population due to the stated recruitment procedures. The sample size was also small and consisted of mainly women. It should be noted that the aim of this study was not to make generalizations about the population from which the sample was drawn, but rather to explore the views of clients attending a community based LMP.

## 5. Conclusions

Effective lifestyle modification programs in routine clinical care and community setting have enormous potential in response to the increasing public health problem of obesity and related diseases. This study demonstrates that clients valued the positive impacts on their health and nutrition knowledge, and the nutritionists’ efforts offered in a community based lifestyle modification program. The results also imply that the nutritionist’s capability of providing quality consultations and patient-centered care is important for empowering clients to achieve lifestyle modification.

## Figures and Tables

**Table 1.** Participant characteristics.

**Characteristics**	**n**	**%**

Age		

Below 20 years	1	4
20–39 years	9	36
40 + years	15	60

Gender		

Male	4	16
Female	21	84

Race/Ethnicity		

Chinese	24	96
Non-Chinese	1	4

Duration since joining the LMP		

Less than 6 months	16	64
Between 6 and 12 months	3	12
More than 12 months	6	24

Tried to lose weight before		

Yes	17	68
No	8	32

BMI at the start of LMP^1^		

<25 kg/m^2^	5	20
≥25 kg/m^2^	15	80

BMI at the time of interview^1^		

<25 kg/m^2^	11	44
≥25 kg/m^2^	14	56

1 Overweight is defined as BMI between 23 and 24.9 kg/m^2^, and obese is defined as BMI of ≥ 25 kg/m^2^ based on the World Health Organization’s criteria for Asians [21]

**Table 2.** Themes identified by researchers from analysis of transcribed interviews.

**Themes**	**Subthemes**
1. Perceived impact of the LMP	On health and satiety On nutrition knowledge and self-control of food intake
2. Perceived barriers to change	Psychological factors Environmental factors
3. Client’s perceptions of nutritionist’s roles in the LMP	Nutritionist being an information source in the LMP Nutritionist being a psychological supporter in the LMP
4. Improvements for the LMP	Provision of longer consultation time Minimize nutritionists’ variations in counseling skill
